# Supplementary material for: Identification of potential vulnerable points and paths of contamination in the Dutch broiler meat trade network
Source: PLoS One. 2020 May 15;15(5):e0233376. doi: 10.1371/journal.pone.0233376 (PMC7228058; doi:10.1371/journal.pone.0233376)
Supplement: S1 File — (DOCX) [file pone.0233376.s001.docx]

# Supplement

## *Graph Theory*

Graph theory is a mathematical method to study the relationships among pairs of actors. A network can be shown as a graph, G=(V,E), that has nodes, V, and connecting edges, E. The graph can be directed graph or unredirected graph. In a directed graph the edges are directed from one start node towards the target node. In contrast, in undirected graph, no differentiation is made between start and end node for the edges. In a trade network model, the nodes represent actors and the edges represent the trade relations between a pair of actors [14, 33-35].

## *Network types*

The network can be centralized or decentralized (see Fig *S1*). A centralized network usually consists of many star structures including star nodes and satellite nodes. Star nodes are the most critical nodes that particularly need focus [34, 36]. In a decentralized network, there are no star nodes; instead the nodes in usually have equal importance [34].

| Fig S1. Two categories of network [34]. |
| --- |

In a centralized network, the critical nodes in the graph can be identified scientifically and precisely by quantifying their degree and betweenness centrality. The edge density of the network represents the trade density of the network. The principle of degree and betweenness centrality, and trade density are introduced as follows.

## Degree centrality

Degree centrality of a node is a measure of how connected the node is to other nodes. It can be divided into in-degree centrality and out-degree centrality. The in- and out-degree centrality are calculated as the number of incoming arcs and outgoing arcs, respectively, of the node. Consider a node *k*, and if there is a trade link from node *k* to a customer node *j*, this trade link counts as one out-degree centrality of node *k*. Similarly, if there is a trade link from a supplier node *i* to the node *k*, this trade link counts as one in-degree centrality of node *k*. The in- and out-degree centrality of node *k* is calculated by counting the its incoming and outgoing arcs, respectively [37]. The out-and-in degree centrality of node k are given by the *equation S1* and *S2* respectively:

|  | $D_{out}\left( k \right)=\sum_{j=1}^{n} a_{kj}$ | (S1) |
| --- | --- | --- |
|  | $D_{in}\left( k \right)=\sum_{i=1}^{n} a_{ik}$ | (S2) |

where *D_out_(k)* is the out-degree of node *k*, *D_in_(k)* is the in-degree of node *k, i* is a supplier node, *j* is a customer node, *k* is the current node, *n* is the total number of nodes, *a_kj_* is trade relation between any potential customer *j* (*j* ≠ *k*) and the current node *k* (if node j is a customer to *k,* then then *a_kj_ =1*; otherwise *a_kj_* =0), *a_ik_* is trade relation between any potential supplier node *i* (*j* ≠ *k*) and the current node *k* (if node *i* is a supplier to *k*, then then *a_ik_* =1; otherwise *a_ik_* =0).

In the following we use the simple example network shown in Fig S2 to demonstrate here the computation of degree centrality, and later on betweenness centrality and trade density. The network including 5 nodes (A, B, C, D, and E) and 7 edges (AB, AD, AE, BC, DC, EB, and EC).

| Fig S2. An example network of a graph with five nodes and seven edges |
| --- |

Node A has three out-going arcs and no in-coming arcs. Therefore, node A’s out-degree centrality is 3 and its in-degree centrality is 0. Likewise, the in- and out-degree centrality of the nodes B, C, D and E are (1, 2), (0, 3), (1, 1) and (2, 1), respectively. Node A has the highest out-degree centrality, and thus it has the highest possibility to spread contaminated products. Node C has the highest in-degree centrality, and likewise, it has the highest possibility of receiving contaminated products.

## Betweenness centrality

Betweenness centrality is used to judge the centrality in undirect graph. It is calculated based on shortest paths, *i.e.*, it indicates how often each node appears on a shortest path between any two nodes (s and t) in the graph. The betweenness centrality of node k is given in equation *S3*. If the betweenness centrality of an actor is higher, more trade paths pass through this actor and it can be seen as a central node that is prone to contamination spread [14].

|  | $G\left( k \right)=\sum_{s\neq k\neq t} \frac{\alpha_{st} (k)}{\alpha_{st}}$ | (S3) |
| --- | --- | --- |

where *G(k)* is betweenness centrality of the current node *k*, *s* and *t* are any two random nodes other than node *k*, *α_st_* is the total number of shortest paths from node *s* to node *t*, and *α_st_(k)* is the number of those paths that are passing through node *k.*

When calculating the betweenness centrality of node A of Fig *S2*, nodes *s* and *v* could be the combination of any two nodes among the nodes B, C, D, and E. The arrows of the edges have no significance for calculating betweenness centrality. Simple calculation will show that betweenness centrality of the nodes A, B, C, D and E are 1, 0.33, 1, 0.33 and 0.33, respectively. The betweenness centrality of node A and C are highest, and according to betweenness centrality value, these two nodes are equally important for contamination spread.

## Trade density

Trade density is the relative measure of edge density of a directed graph that can be used to analyse the total complexity of a network. It is calculated by the ratio of existing number of edges to all possible edges between the nodes in the network. If the trade density is high, the trade relations is more complex and the spread of contamination cannot be controlled easily [14]. Trade density is calculated by using equation *S4*.

|  | $D= \frac{\vert E\vert}{\vert V\vert(\left\vert V \right\vert-1)}$ | (S4) |
| --- | --- | --- |

where *D* is the relative edge density of the directed graph, *E* is the number of edges in the graph, and *V* is the number of nodes in the graph.

The calculation of trade density is straight forward. For instance, the network shown in Fig S2 has 5 nodes and 7 directed edges. Its trade density is, therefore, 35% (=7/(5*(5-1))).

## Gravity model

Generally, gravity modelling is used to estimate the likelihood of a trade connection between any two actors in a trade network when trade linkages are unknown or missing. The gravity model calculates the probability of the existence of trade relation between to nodes, which is proportional to the capacity of the involved actors and is inversely proportional to the geographical distance between actors. The probability has to be computed for every pair of nodes. If the possibility is larger than 0.5, we can assume a trade link between the actors; otherwise, we assume that there is no trade link (Bergeijk and Brakman 2010; Kepaptsoglou, Karlaftis, and Tsamboulas 2010). The trade link between a potential supplier *i* and a potential customer *j* is estimated using equation *S5*.

|  | $q\left( i, j \right)=q_{0} \frac{c(i)c(j)}{d(i,j)}$ | (S5) |
| --- | --- | --- |

where *q(i,j)* is the possibility of trade relation between a supplier node *i* and a customer node *j*, *c(i)* is the capacity of supplier *i, c(j)* is the capacity of customer *j*, *d(i,j)* is the distance between supplier *i* and customer *j*, *q_0_* is a scaling factor.

The value of *q(i,j)* should lie between 0 and 1. The scaling factor *q_0_* ensures that *q(i,j)* will be between 0 and 1 and estimated trade lines match the known trade links from the available data.
