# Supplementary material for: Identification of potential vulnerable points and paths of contamination in the Dutch broiler meat trade network
Source: PLoS One. 2020 May 15;15(5):e0233376. doi: 10.1371/journal.pone.0233376 (PMC7228058; doi:10.1371/journal.pone.0233376)
Supplement: S1 Data — (ZIP) [file pone.0233376.s002.zip › processors_list.docx]

|  | Name | Location | Capacity | Raw | Processed | Cutting | Impot/export | Year | Source |
| --- | --- | --- | --- | --- | --- | --- | --- | --- | --- |
| 1 | 2sistersstorteboom | Kornhorn | 395.2 million st/year | X |  | X |  | 2017 | 1 |
| 2 | 2sistersstorteboom | Nijkerk | 4million st/week | X | X |  |  | 2018 | 2 |
| 3 | 2sistersstorteboom | Kotowo | 4million st/week | X | X |  | Poland | 2018 | 2 |
| 4 | Friki (from Plukon) | Wezep | - |  | X |  |  |  | 3 |
| 5 | STARA FOODS BV | Gervenseweg | - |  |  |  |  |  | 4 |
| 6 | G.P.S. Nunspeet | **Nunspeet** | $215.95M | X |  | X |  |  | 5 |
| 7 | Frisia Food BV | Haulerwijk | $ 92.24M | X |  | X | Africa, Bulgaria, China, Germany, England, Finland, France, Malaysia, Norway, Poland, Rumania and Russia. | 2018 |  |
| 8 | v.d. Bor Pluimveeslachterij | Nijkerkerveen | $26.25M | X |  | X |  |  |  |
| 9 | [Ven Poultry B.V.](http://www.hoovers.com/company-information/cs/company-profile.ven_poultry_bv.a0d5525088ae3d9f.html) | Lierop | $4.18M | X |  |  |  |  |  |
| 10 | [J. Tromp](http://www.hoovers.com/company-information/cs/company-profile.j_tromp.bd7cf478ba138a42.html) | Purmerland | $0.92M | X | X | X |  |  |  |
| 11 | [Nijhof Nijkerk B.V.](http://www.hoovers.com/company-information/cs/company-profile.nijhof_nijkerk_bv.35066bae0a3914ea.html) | Nijkerk gld | $0.65M | X | X | X |  |  |  |
| 12 | [Khumex Poultry Products BV](http://www.hoovers.com/company-information/cs/company-profile.khumex_poultry_products_bv.f73389b09d7aa85e.html) | Zeewolde | 400tonne/week | X |  | X | Great Britain  France  Belgium  Ireland  Germany |  |  |
| 13 | Poeliersbedrijf Ep de Graaf | Putten | 2000pallets | X | X | X | Belgium and Germany |  |  |
| 14 | [CFG Poultry B.V.](http://www.hoovers.com/company-information/cs/company-profile.cfg_poultry_bv.7c4cf214db17ed58.html) | Nijkerk gld, | 2million consumer/week | X | X | X | EU |  |  |
| 15 | [Wijnen Meat B.V.](http://www.hoovers.com/company-information/cs/company-profile.wijnen_meat_bv.5cb893d0a7fcd99c.html) | Oss | 600tonnes/week |  | X |  |  |  |  |
| 16 | W. van der Meer en Zonen | Dronrijp | 0.125million st/week | X | X | X |  |  |  |
| 17 | Poeliersbedrijf Van Berkel | Loon op zand | - | X | X | X |  |  |  |
| 18 | [Polskamp B.V.](http://www.hoovers.com/company-information/cs/company-profile.polskamp_bv.d42af1681b482630.html) | Ermelo | 5.000 tons meats | X |  | X |  | 01 |  |
| 19 | [Polskamp B.V.](http://www.hoovers.com/company-information/cs/company-profile.polskamp_bv.d42af1681b482630.html) | Harskamp | 5.000 tons meats | X |  | X |  | 01 |  |
| 20 | [Poelier van der Wal B.V.](http://www.hoovers.com/company-information/cs/company-profile.poelier_van_der_wal_bv.1fe6bfb78b623fdf.html) |  | - | X |  | X |  |  |  |
| 21 | [Mr. John Chicken Food Systems B.V.](http://www.hoovers.com/company-information/cs/company-profile.mr_john_chicken_food_systems_bv.01ceb24b2e2cb88c.html) | Zevenhuizen | $0.31M |  | X |  |  |  |  |
| 22 | TD Poultry Processing BV | Veenendaal | - |  |  |  | EU, Brazil, Argentina and Chile, |  |  |
| 23 | [Van Loon Son B.V.](http://www.hoovers.com/company-information/cs/company-profile.van_loon_son_bv.f30d053b42758d09.html) | Son en breugel | $320.62M |  | X | X |  |  | 6 |
| 24 | [N. Vriesekoop B.V.](http://www.hoovers.com/company-information/cs/company-profile.n_vriesekoop_bv.7f2067005f0f67e4.html) | Zoetermeer | $215.94M | X |  | X | UK, Irland |  |  |
| 25 | [Van Hessen B.V.](http://www.hoovers.com/company-information/cs/company-profile.van_hessen_bv.50af663ee3729321.html) | Nieuwerkerk | $138.26M | X | X |  |  |  |  |
| 26 | Heijs Food Products | Hoogeveen | $102.18M | X |  | X |  |  |  |
| 27 | [Albert van Zoonen B.V.](http://www.hoovers.com/company-information/cs/company-profile.albert_van_zoonen_bv.652bda0dad901529.html) | Schagen | $12.04M | X | X | X |  |  |  |
| 28 | [Vleesbedrijf Bolscher](http://www.hoovers.com/company-information/cs/company-profile.vleesbedrijf_bolscher_bv.891724f3bac568b3.html) | Enschede | $4.58M (ALL) |  | X |  |  |  |  |
| 29 | [Thyker B.V.](http://www.hoovers.com/company-information/cs/company-profile.thyker_bv.344d95273296962b.html) | Barneveld | $1.83M | X | X | X | EU |  |  |
| 30 | [Horeca Specialiteiten Giessen](http://www.hoovers.com/company-information/cs/company-profile.horeca_specialiteiten_giessen.2384cdf59974951d.html) | Wijk en aalburg | $0.91M |  | X |  |  |  |  |
| 31 | [Vee- en Vleeshandel Verschoor B.V.](http://www.hoovers.com/company-information/cs/company-profile.vee-_en_vleeshandel_verschoor_bv.380d8e681528672a.html) | Leiderdorp | $0.9M | X |  | X |  |  |  |
| 32 | [Atlas Halal Products B.V.](http://www.hoovers.com/company-information/cs/company-profile.atlas_halal_products_bv.ad62eb406f814bce.html) | Montfoort | $0.57M |  | X |  |  |  |  |
| 33 | [JP puurvlees](http://www.hoovers.com/company-information/cs/company-profile.jp_puurvlees.4c91d54caf83183f.html) | Nunspeet | $0.35M | X |  | X |  |  |  |
| 34 | [Biologische Boerderij Sumiran](http://www.hoovers.com/company-information/cs/company-profile.biologische_boerderij_sumiran.576df3140cfbf9ce.html) | Heusden gem | $0.27M | X |  | X |  |  |  |
| 35 | [J.P. Gringhuis](http://www.hoovers.com/company-information/cs/company-profile.jp_gringhuis.422f74e06de95bf0.html) | Emmen | $0.25M | X | X | X |  |  |  |
| 36 | [J. Borgmeier Dienstverlening](http://www.hoovers.com/company-information/cs/company-profile.j_borgmeier_dienstverlening.3e1f85547441d216.html) | Zwolle | $0.25M | X | X | X |  |  |  |
| 37 | [Van der Meer Vlees](http://www.hoovers.com/company-information/cs/company-profile.van_der_meer_vlees.a424f1de6024b371.html) | Eersel | $0.21M |  | X |  |  |  |  |
| 38 | [Quality Halal Meat](http://www.hoovers.com/company-information/cs/company-profile.quality_halal_meat.cac3102445b8f1c1.html) | Rotterdam | $0.19M | X |  | X |  |  |  |
| 39 | [Jandlis Meat](http://www.hoovers.com/company-information/cs/company-profile.jandlis_meat.ae4607481aa13e6b.html) | Dronten | $0.17M | X |  | X |  |  |  |
| 40 | [Pluimveeslachterij De Wolf](http://www.hoovers.com/company-information/cs/company-profile.pluimveeslachterij_de_wolf.34eef3d91d6deda8.html) | Dongen | $0.15M | X |  | X |  |  |  |
| 41 | Maassen Eieren Homoet | Homoet | Valburg, Bemmel, Arnhem, Driel, Heteren, Elst, Herveld, Gendt, Ressen, Nijmegen | X |  |  |  |  | 7 |
| 42 | Bilal Chicken Centre | Amsterdam | 0.1millionst/week |  | X |  |  |  | 8 |
| 43 | pingo poultry | Wezep | 6.5 million/week  €740 million | X |  |  | France, Belgium, Germany |  | 9 |
| 44 | Devries | Nijkerkerveen | 150tonnes/week | X |  | X | Belgium, German, English and Irish |  | 10 |
| 45 | GroenlandKip | Bodegraven | €2500 million |  | X |  |  |  | 11 |
| 46 | kipkruidennl-henny-penny | Nieuwerkerk aan den ijssel | Order |  | X |  |  |  | 12 |
| 47 | Emin Chicken BV | Amsterdam | Order |  | X |  |  |  | 13 |
| 48 | Koylu Food | Eindhoven | Order |  | X |  |  |  | 14 |
| 49 | KappersFoods | Cuijk | Order |  | X |  |  |  | 15 |
| 50 | Jan Zandbergen | Veenendaal | 20,000 tonnes | X | X |  |  |  | 16 |
| 51 | H. Ferwerda BV | Rotterdam | 3000 tonnes | X |  |  |  |  | 17 |
| 52 | Ferdinand Zandbergen B | Woudenberg | 1000ton/week | X | X |  |  | 02 | 18 |
| 53 | Group of Butchers | Eersel | € 6787million/year |  | X |  |  |  | 19 |
| 54 | Group of Butchers | Alken | € 6787million/year |  | X |  |  |  |  |
| 55 | Group of Butchers | Oosterzele | € 6787million/year |  | X |  |  |  |  |
| 56 | Group of Butchers | Barneveld | € 6787million/year |  | X |  |  |  |  |
| 57 | Group of Butchers | Tiburg | € 6787million/year |  | X |  |  |  |  |
| 58 | Van Loon Vlees | Eersel | € 3251million/year |  | X |  |  |  |  |

1 World`s top companies profile of the world`s 289 leading poultry companies. Poultry International. <http://www.poultryinternational-digital.com/201710/index.php?startid=22#/1>

2 <https://www.2sistersstorteboom.nl/over-ons>

3 <http://www.friki.nl/uit-een-goed-nest/>

4 <https://www.starafoods.com/#nav-history>

5 <http://www.hoovers.com/company-information/company-search.html?term=chicken&nvcnt=72&maxitems=100&sortDir=Descending&sort=SalesUS>

01 <https://www.youtube.com/watch?v=M9ES-XTmmG4>

02 <https://www.kompass.com/z/nl/c/jan-zandbergen-bv/nl209520/>

| No. | Name of comapny | Location | Sales |
| --- | --- | --- | --- |
|  |  |  |  |
| 1 | 2sistersstorteboom | Nijkerk | 436 M € |
| 2 | 2sistersstorteboom | Kotowo | 436 M € |
| 3 | Friki (from Plukon) | Wezep | >500 M |
| 4 | STARA FOODS BV | Gervenseweg | —— |
| 5 | [Ven Poultry B.V.](http://www.hoovers.com/company-information/cs/company-profile.ven_poultry_bv.a0d5525088ae3d9f.html) | Lierop | $4.18M |
| 6 | Poeliersbedrijf Ep de Graaf | Putten | 2000pallets |
| 7 | [CFG Poultry B.V.](http://www.hoovers.com/company-information/cs/company-profile.cfg_poultry_bv.7c4cf214db17ed58.html) | Nijkerk gld, | 2million consumer/week |
| 8 | [Wijnen Meat B.V.](http://www.hoovers.com/company-information/cs/company-profile.wijnen_meat_bv.5cb893d0a7fcd99c.html) | Oss | 600tonnes/week |
| 9 | [Mr. John Chicken Food Systems B.V.](http://www.hoovers.com/company-information/cs/company-profile.mr_john_chicken_food_systems_bv.01ceb24b2e2cb88c.html) | Zevenhuizen | $0.31M |
| 10 | TD Poultry Processing BV | Veenendaal | —— |
| 11 | [Van Hessen B.V.](http://www.hoovers.com/company-information/cs/company-profile.van_hessen_bv.50af663ee3729321.html) | Nieuwerkerk | $138.26M |
| 12 | [Vleesbedrijf Bolscher](http://www.hoovers.com/company-information/cs/company-profile.vleesbedrijf_bolscher_bv.891724f3bac568b3.html) | Enschede | $4.58M |
| 13 | [Horeca Specialiteiten Giessen](http://www.hoovers.com/company-information/cs/company-profile.horeca_specialiteiten_giessen.2384cdf59974951d.html) | Wijk en aalburg | $0.91M |
| 14 | [JP puurvlees](http://www.hoovers.com/company-information/cs/company-profile.jp_puurvlees.4c91d54caf83183f.html) | Nunspeet | $0.35M |
| 15 | [Van der Meer Vlees](http://www.hoovers.com/company-information/cs/company-profile.van_der_meer_vlees.a424f1de6024b371.html) | Eersel | $0.21M |
| 16 | Maassen Eieren Homoet |  | 知道 |
| 17 | Bilal Chicken Centre | Amsterdam | €1.5million |
| 18 | pingo poultry | Wezep | €370 million |
| 19 | GroenlandKip | Bodegraven | $138.26M |
| 20 | kipkruidennl-henny-penny | Nieuwerkerk aan den ijssel | Order |
| 21 | Emin Chicken BV | Amsterdam | Order |
| 22 | Koylu Food | Eindhoven | € 0.4M |
| 23 | KappersFoods | Cuijk | €300 million |
| 24 | Jan Zandbergen | Veenendaal | €300 million |
| 25 | H. Ferwerda BV | Rotterdam | 45M |
| 26 | Ferdinand Zandbergen B | Woudenberg | €300 million |
|  | Marel Stork Poultry Processing BV | Dongen | 514 |
|  | Marel Stork Poultry Processing BV | Boxmeer | 514 |
|  | [Poelier van der Wal B.V.](http://www.hoovers.com/company-information/cs/company-profile.poelier_van_der_wal_bv.1fe6bfb78b623fdf.html) | Kollumerzwaag |  |
|  | [Broviand B.V.](http://www.hoovers.com/company-information/cs/company-profile.broviand_bv.ee7bd914ba123ff8.html) | Someren | 300 |
|  | [Nijhof Nijkerk B.V.](http://www.hoovers.com/company-information/cs/company-profile.nijhof_nijkerk_bv.35066bae0a3914ea.html) | Nijkerk Gld | $0.65M |
|  | [Firma Poelierderij Jo van Druenen](http://www.hoovers.com/company-information/cs/company-profile.firma_poelierderij_jo_van_druenen.badafb370c3e0d50.html) |  | 0.48 |
