# Supplementary material for: Identification of potential vulnerable points and paths of contamination in the Dutch broiler meat trade network
Source: PLoS One. 2020 May 15;15(5):e0233376. doi: 10.1371/journal.pone.0233376 (PMC7228058; doi:10.1371/journal.pone.0233376)
Supplement: S1 Data — (ZIP) [file pone.0233376.s002.zip › code_betweenness_centrality.pdf]

Betweenness Centrality

Contents

003 Relation hacthery broiler 2
Betweenness Centrality . . . 2
Network Visualization . . . 3

004 Relation broiler-slaughterhouse 4
Betweenness Centrality . . . 4
Network Visualization . . . 5

005 Relation broiler-processor 6
Betweenness Centrality . . . 6
Network Visualization . . . 7

006 Relation Slaugtherhouse-processor 8
Betweenness Centrality . . . 8
Network Visualization . . . 9

007 Relation Processor Retailer 10
Betweenness Centrality . . . 10
Network Visualization . . . 11

008 Slaughterhouse Importer Exporter 12
Betweenness Centrality . . . 12
Network Visualization . . . 13

009 Processor Importer Exporter 14
Betweenness Centrality . . . 14
Network Visualization . . . 15

All Cities 16
Betweenness Centrality . . . 16
Network Visualization (First 20 Cities) . . . 17
Network Visualization . . . 18

Network R code 19

003 Relation hacthery broiler

Betweenness Centrality

ImportantCity	BetweennessScore
Staphorst_C023	6557.04
Hof van Twente_C023	5698.80
Bernheze_C023	2721.23
Stadskanaal_D345	2204.05
Ede_C023	1840.52
Dalfsen_C023	1731.07
Heerhugowaard_D345	1524.22
Utrechtse Heuvelrug_C023	1294.56
Eersel_C023	1167.89
Mill en Sint Hubert_D345	1110.76
Borsele_D345	1088.16
Hardenberg_C023	1008.08
Raalte_D345	910.56
Oss_C023	823.28
Doetinchem_D345	809.31
Achtkarspelen_D345	806.96
Ooststellingwerf_D345	779.31
Sint Anthonis_C023	562.94
Eersel_D345	543.71
Midden-Drenthe_C023	538.11

Network Visualization

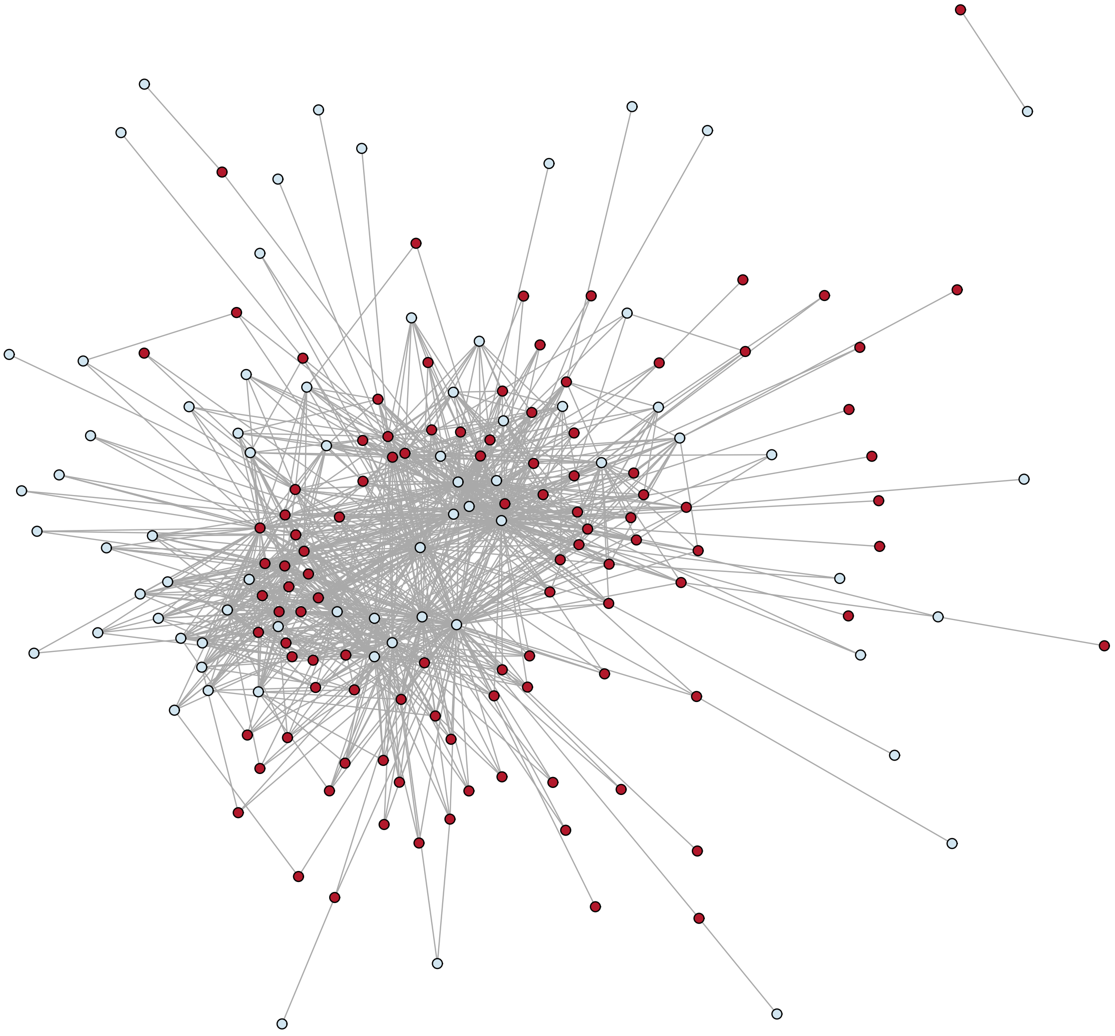

Figure 1: Network

#B2182B: D345, **#D1E5F0:** C023

004 Relation broiler-slaughterhouse

Betweenness Centrality

ImportantCity	BetweennessScore
Nijkerk_3_E468	4013.98
Grootegast._E468	3155.01
Zuidplas_E468	702.96
Someren_D345	698.32
Hardenberg_D345	667.49
Peel en Maas_D345	632.94
Oldebroek_E468	575.01
Hof van Twente_E468	560.67
Nunspeet_E468	381.45
Stichtse Vecht_E468	303.58
Midden-Drenthe_D345	294.50
Zeewolde_E468	292.01
Leudal_D345	268.36
Deventer_D345	241.66
Doetinchem_E468	230.15
Borger-Odoorn_D345	218.59
Putten_E468	216.57
Barneveld_1_E468	208.79
Ede_D345	201.52
Hof van Twente_D345	180.28

Network Visualization

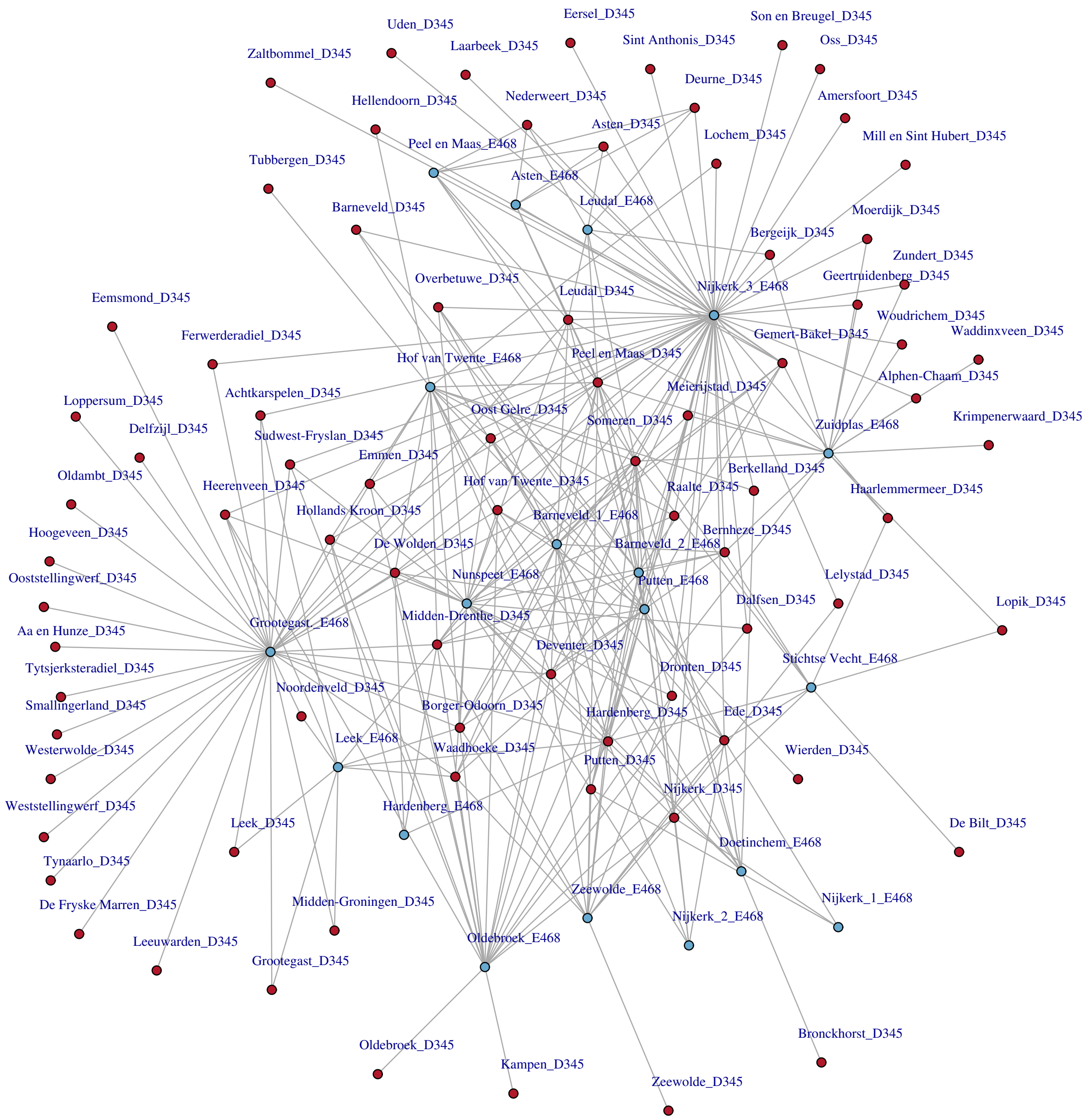

Figure 2: Network

#B2182B: D345, **#67A9CF:** E468

005 Relation broiler-processor

Betweenness Centrality

ImportantCity	BetweennessScore
Hoogeveen_F5679	5004.998
Son en Breugel_F5679	4623.488
Zoetermeer_F5679	4351.310
Eersel_1_F5679	4293.488
Ede_F5679	2776.884
Ermelo_F5679	2747.599
Ooststellingwerf_F5679	1570.728
Aalten_D345	404.319
Emmen_D345	338.803
Cranendonck_D345	330.028
Waadhoeke_F5679	314.235
Hoogeveen_D345	163.566
Buren_D345	145.038
Bergeijk_D345	122.934
Dalfsen_D345	122.934
Borger-Odoorn_D345	114.556
Echt-Susteren_D345	100.410
Delfzijl_D345	92.690
Deurne_D345	83.664
Bronckhorst_F5679	29.071

Network Visualization

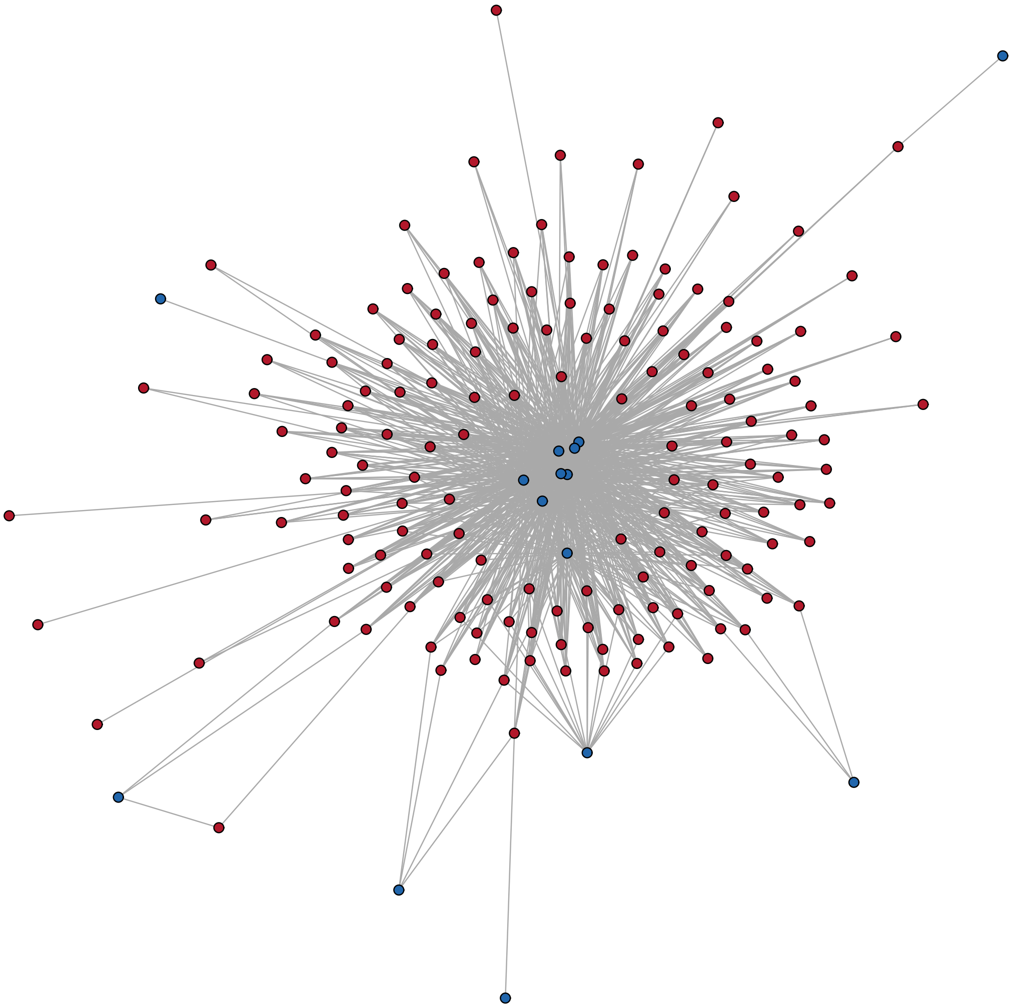

Figure 3: Network

#B2182B: D345, **#2166AC:** F5679

006 Relation Slaugtherhouse-processor

Betweenness Centrality

ImportantCity	BetweennessScore
Nijkerk_3_E468	223.917
Zuidplas_E468	105.784
Doetinchem_E468	93.220
Barneveld_1_E468	56.240
Barneveld_2_E468	56.240
Putten_E468	56.240
Best_F5679	51.668
Midden-Drenthe_F5679	51.668
Roosendaal_F5679	51.668
Oss_F5679	51.668
Zuidplas_1_F5679	51.668
Bodegraven_F5679	51.668
Cuijk_F5679	51.668
Veenendaal_F5679	51.668
Rotterdam_F5679	51.668
Woudenberg_F5679	51.668
Someren_1_F5679	51.668
Someren_F5679	41.063
Nunspeet_E468	38.253
Zeewolde_E468	38.253

Network Visualization

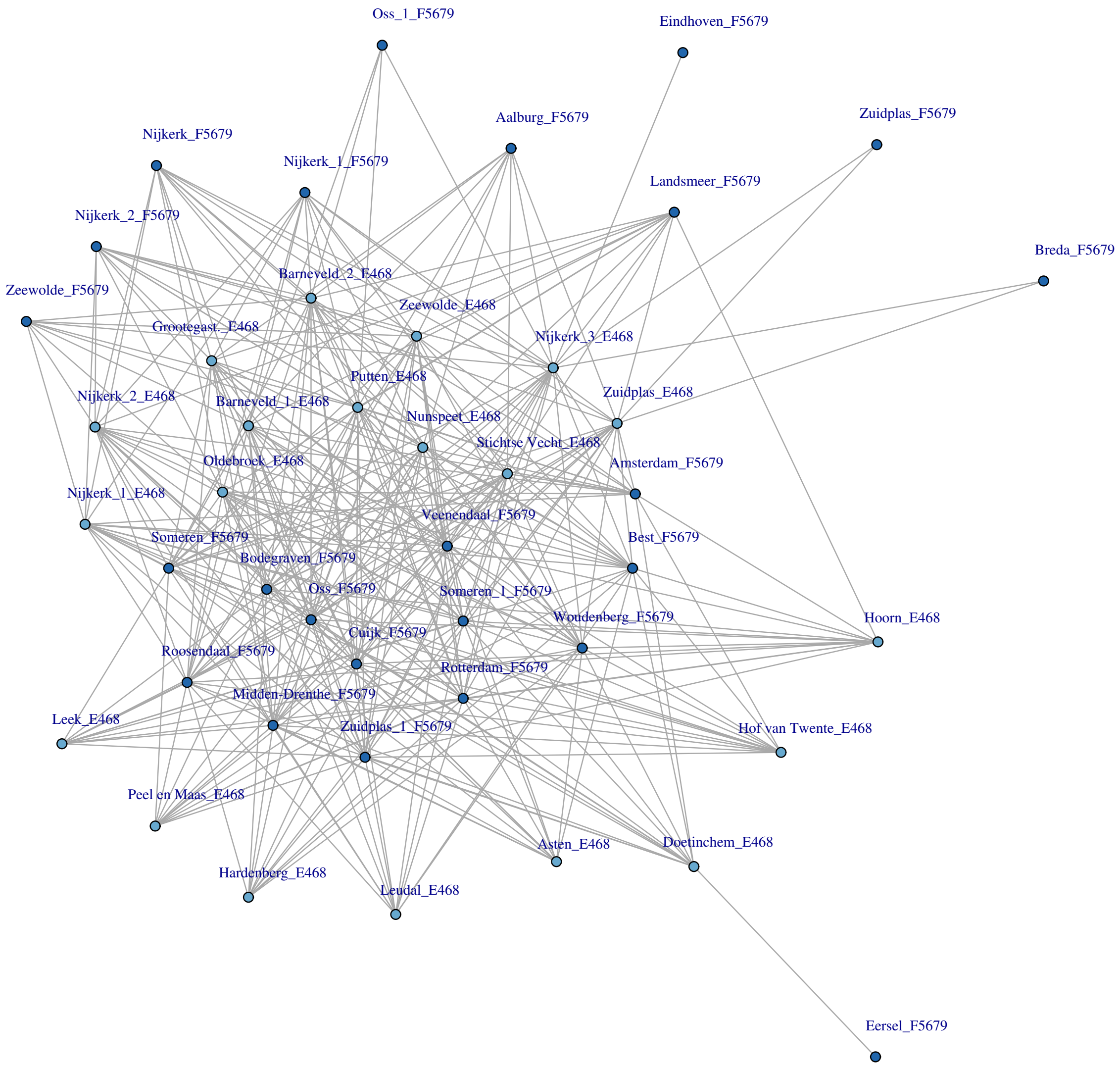

Figure 4: Network

#67A9CF: E468, **#2166AC:** F5679

007 Relation Processor Retailer

Betweenness Centrality

ImportantCity	BetweennessScore
Hoogeveen_F5679	10980.6
Cuijk_F5679	10430.3
Veenendaal_F5679	10430.3
Zoetermeer_F5679	10152.6
Someren_1_F5679	9948.1
Son en Breugel_F5679	9948.1
Eersel_1_F5679	9948.1
Ede_F5679	9468.8
Bodegraven_F5679	9368.0
Ermelo_F5679	9324.1
Zuidplas_1_F5679	9304.7
Woudenberg_F5679	9200.2
Roosendaal_F5679	8006.6
Ooststellingwerf_F5679	7579.3
Oss_F5679	7560.1
Rotterdam_F5679	5878.4
Best_F5679	5460.3
Midden-Drenthe_F5679	3329.3
Amsterdam_G007	1785.9
Waadhoeke_F5679	1670.1

Network Visualization

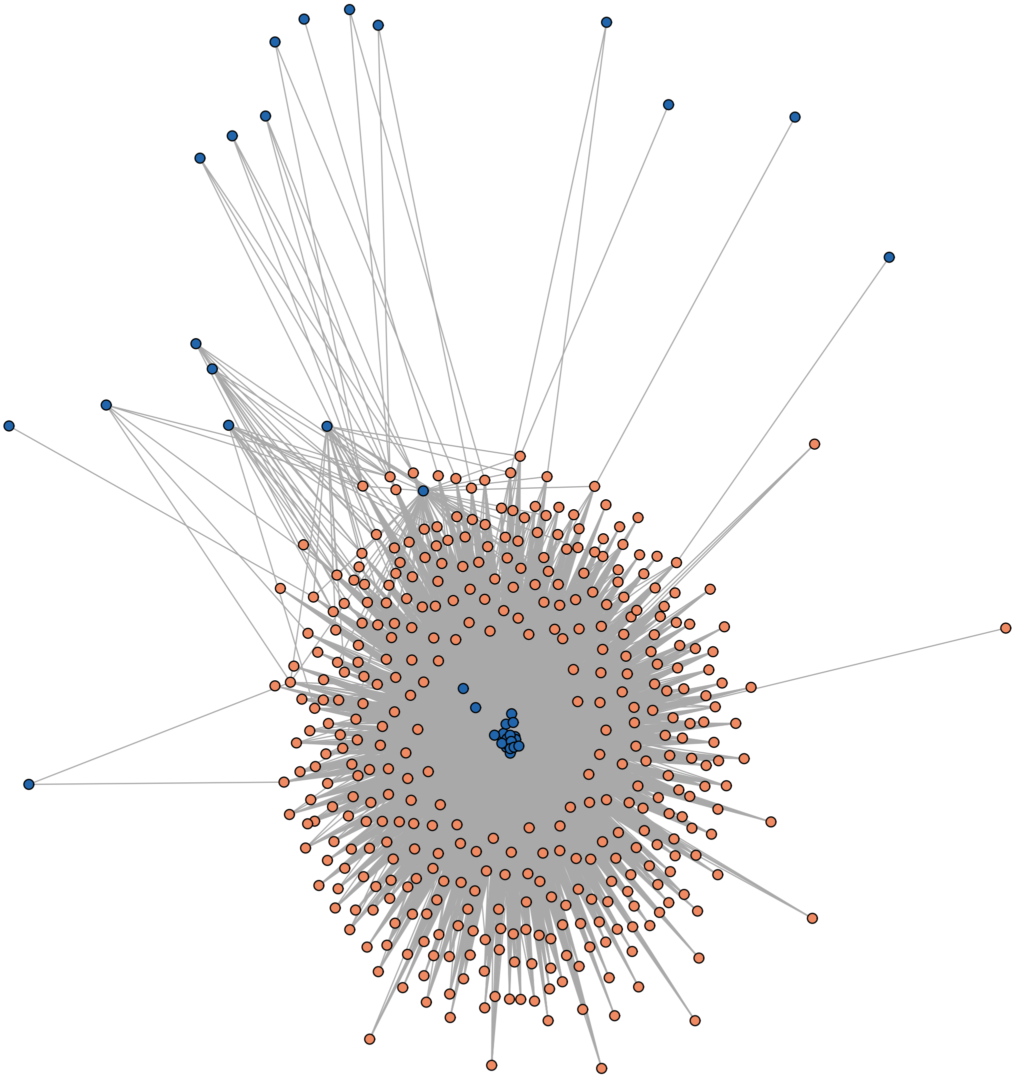

Figure 5: Network

#EF8A62: G007, **#2166AC:** F5679

008 Slaughterhouse Importer Exporter

Betweenness Centrality

ImportantCity	BetweennessScore
Importer__H009	183
Exportert__H009	15
Barneveld__1__E468	3
Putten__E468	3
Grootegast.__E468	3
Nijkerk__1__E468	3
Nijkerk__2__E468	3
Zeewolde__E468	3

Network Visualization

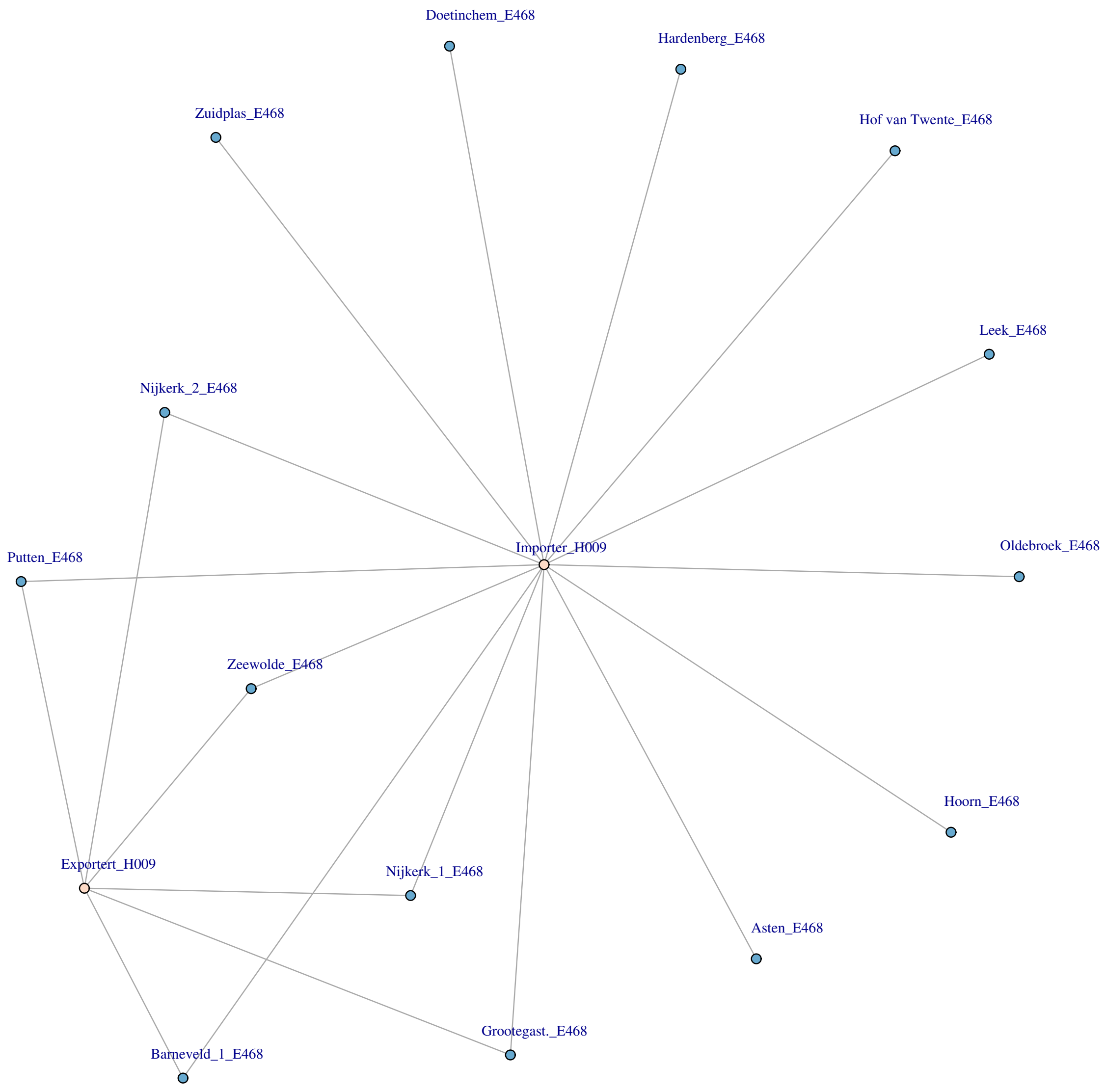

Figure 6: Network

#FDDBC7: H009, **#67A9CF:** E468

009 Processor Importer Exporter

Betweenness Centrality

ImportantCity	BetweennessScore
Importer_H009	216
Exportert_H009	36
Zeewolde_F5679	11
Someren_F5679	11
Zuidplas_1_F5679	11
Kollumerland en Nieuwkruisland_F5679	11

Network Visualization

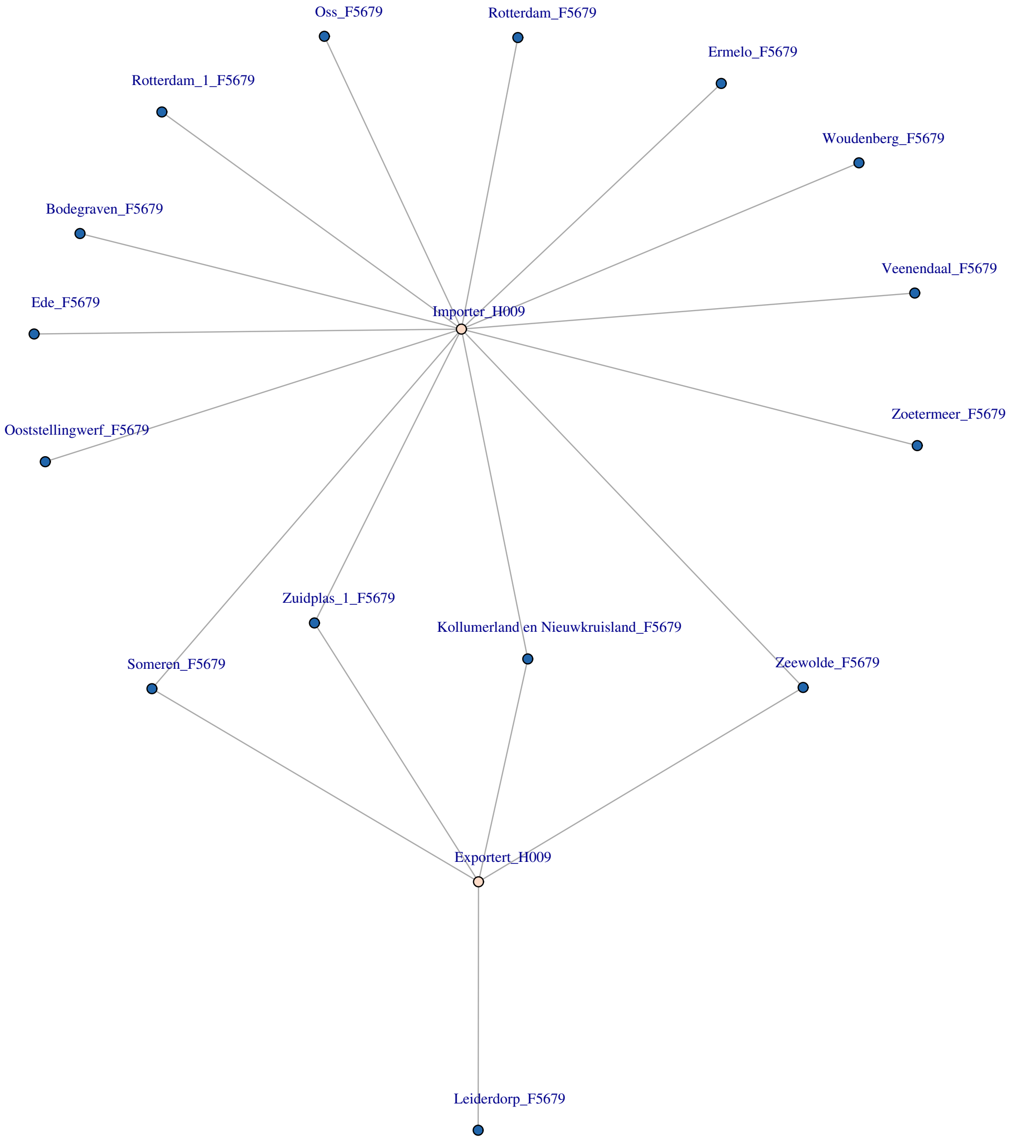

Figure 7: Network

#FDDBC7: H009, **#2166AC:** F5679

All Cities

Betweenness Centrality

ImportantCity	BetweennessScore
Hoogeveen_F5679	52270.6
Zoetermeer_F5679	50062.3
Son en Breugel_F5679	49657.7
Eersel_1_F5679	48538.2
Ede_F5679	41363.4
Ermelo_F5679	40065.4
Ooststellingwerf_F5679	29646.2
Zuidplas_1_F5679	11242.7
Veenendaal_F5679	11242.4
Cuijk_F5679	11092.4
Bodegraven_F5679	10078.5
Woudenberg_F5679	9890.1
Roosendaal_F5679	8466.1
Oss_F5679	8110.3
Stadskanaal_D345	7540.7
Waadhoeke_F5679	6486.8
Rotterdam_F5679	6304.0
Best_F5679	5750.3
Heerhugowaard_D345	5278.1
Staphorst_C023	5224.0

Network Visualization (First 20 Cities)

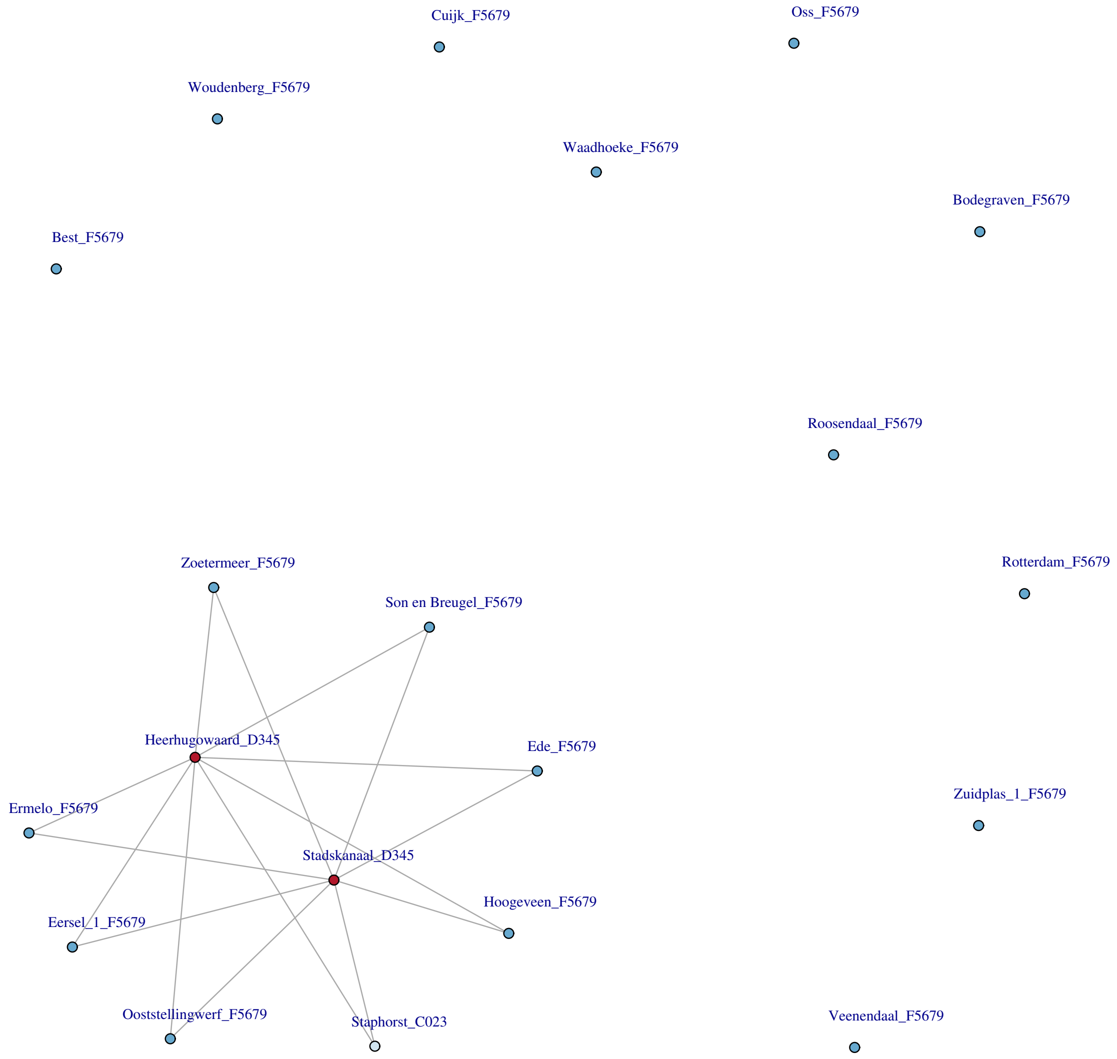

Figure 8: Network

#B2182B: D345, **#D1E5F0:** C023, **#67A9CF:** F5679

Network Visualization

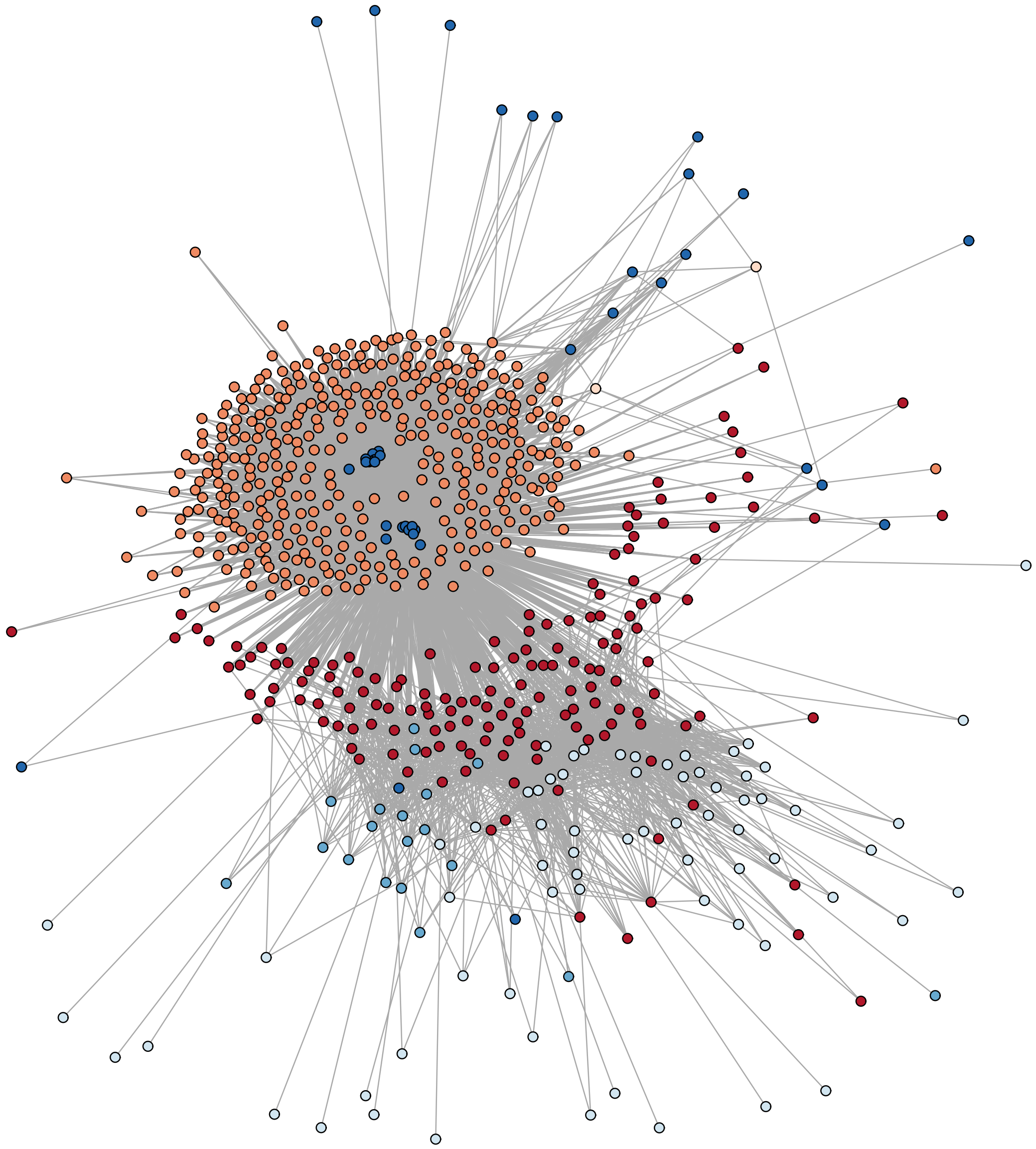

Figure 9: Network

#B2182B: D345, **#EF8A62:** G007, **#FDDBC7** H009, **#D1E5F0:** C023, **#67A9CF:** E468, **#2166AC:** F5679

Network R code

```
require(pacman)
p_load(tidyverse, data.table, sna, plyr, readxl, tibble, RColorBrewer)

Read_Excel <- function(filename){
  # Read excel file
  # Author: Shuai Hao
  # Args:
  #     filename: excel file under the current directory
  # Returns:
  #     data frame
  SheetNames <- excel_sheets(filename)
  SheetName <- SheetNames[SheetNames%in%c("relations", "Relation",
                                          "relation", "relaton")]
  Dat <- read_excel(filename, sheet=SheetName)
  Dat <- Dat[-nrow(Dat), -ncol(Dat)]
  colnames(Dat)[1] <- "rowname"
  Dat <- column_to_rownames(Dat)
  return(Dat)
}


Wide_To_Long <- function(filename){
  # Convert Wide form to Long form
  # Author: Shuai Hao
  # Args:
  #     filename: excel file under the current directory
  # Returns:
  #     data frame with long form
  Relations <- c("relations", "Relation", "relation", "relaton")
  SheetNames <- excel_sheets(filename)
  SheetName_Rel <- SheetNames[SheetNames%in%Relations]
  Rel_Dat <- read_excel(filename, sheet=SheetName_Rel)
  Rel_Dat <- Rel_Dat[-nrow(Rel_Dat), -ncol(Rel_Dat)]
  colnames(Rel_Dat)[1] <- "from"
  Dis_Dat <- read_excel(filename, sheet="distance")
  colnames(Dis_Dat)[1] <- "from"
  Rel_Long <- gather(Rel_Dat, to, Relations, -from)
  Dis_Long <- gather(Dis_Dat, to, Distance, -from)
  Dat <- full_join(Rel_Long, Dis_Long)
  filename <- gsub(".xlsx", "", filename)
  Dat$FileName <- filename
  write.csv(Dat, paste0("Long_", filename, ".csv"), row.names = FALSE)
  return(Dat)
}

Mutate_Network <- function(data, end){
  # Calculate betweenness score and mutate the network data
  # Author: Shuai Hao
  # Args:
  #     data: a data frame
  #     end: the first n cities to choose
  # Returns:
  #     Betweenness score and network data
  Mat1 <- matrix(0, nrow(data), nrow(data),
                 dimnames = list(rownames(data), rownames(data)))
  Mat2 <- matrix(0, ncol(data), ncol(data),
                 dimnames = list(names(data), names(data)))

  NetworkDat <- rbind(cbind(Mat1, data), cbind(t(data), Mat2))
  allz <- which(colSums(NetworkDat)<1)
  if(length(allz)==0){
    NetworkDat <- NetworkDat
  }else{
    NetworkDat <- NetworkDat[-allz, -allz]
  }
```

```r
  Res <- betweenness(NetworkDat)
  City <- rownames(NetworkDat)[order(Res, decreasing = TRUE)]
  CityDat <- data.frame(ImportantCity=City,
                        BetweennessScore=sort(Res, decreasing = TRUE))
  CityDat <- CityDat%>%
    filter(BetweennessScore!=0)
  return(list(NetworkDat, CityDat[1:end, ]))
}

filenames <- dir(pattern=".xlsx")
ResList1 <- llply(filenames, Read_Excel)

names(ResList1[[1]]) <- paste0(names(ResList1[[1]]), "_C023")
rownames(ResList1[[1]]) <- paste0(rownames(ResList1[[1]]), "_D345")

names(ResList1[[2]]) <- paste0(names(ResList1[[2]]), "_E468")
rownames(ResList1[[2]]) <- paste0(rownames(ResList1[[2]]), "_D345")

names(ResList1[[3]]) <- paste0(names(ResList1[[3]]), "_F5679")
rownames(ResList1[[3]]) <- paste0(rownames(ResList1[[3]]), "_D345")

names(ResList1[[4]]) <- paste0(names(ResList1[[4]]), "_F5679")
rownames(ResList1[[4]]) <- paste0(rownames(ResList1[[4]]), "_E468")

names(ResList1[[5]]) <- paste0(names(ResList1[[5]]), "_F5679")
rownames(ResList1[[5]]) <- paste0(rownames(ResList1[[5]]), "_G007")

names(ResList1[[6]]) <- paste0(names(ResList1[[6]]), "_E468")
rownames(ResList1[[6]]) <- paste0(rownames(ResList1[[6]]), "_H009")

names(ResList1[[7]]) <- paste0(names(ResList1[[7]]), "_F5679")
rownames(ResList1[[7]]) <- paste0(rownames(ResList1[[7]]), "_H009")

Sep.Net <- llply(ResList1, Mutate_Network, end=20)

# Combine 3, 4
ResList1 <- llply(ResList1, rownames_to_column, var="rowname")
Dat1 <- full_join(ResList1[[1]], ResList1[[2]], by="rowname")

# Combine 5, 6 and 7
Dat2 <- full_join(ResList1[[3]], ResList1[[4]], by="rowname")
Dat3 <- rbind(Dat2, ResList1[[5]][, names(Dat2)])
InterSet <- intersect(names(Dat3), names(ResList1[[7]]))
Dat4 <- rbind(Dat3[, InterSet], ResList1[[7]][, InterSet])
# Combine 1, 2, 3, 4, 5, 6 and 7
Dat5 <- full_join(Dat1, Dat4, by="rowname")
Dat6 <- full_join(Dat5, ResList1[[6]], by="rowname")
Dat6[is.na(Dat6)] <- 0

Dat6 <- column_to_rownames(Dat6)
Dat6 <- Dat6%>%select(-contains("_E468.y"))
names(Dat6) <- gsub("_E468.x", "_E468", names(Dat6))
DuplCity <- intersect(names(Dat6), rownames(Dat6))
Dat6 <- Dat6[!rownames(Dat6)%in%DuplCity, ]
write.csv(Dat6, "CityFullList.csv", row.names=FALSE)
CityDat <- Mutate_Network(Dat6, end=20)

Names <- as.character(CityDat[[2]][, 1])
SubNetworkDat <- CityDat[[1]][rownames(CityDat[[1]])%in%Names,
                              names(CityDat[[1]])%in%Names]

require(igraph)
col=brewer.pal(n = 6, name = "RdBu")
Ecolors <- rep(c(col[1], col[4]), c(106, 70))
G1<-graph_from_adjacency_matrix(as.matrix(Sep.Net[[1]][[1]]), mode="undirected")
tkplot(G1, vertex.size=4,vertex.label.cex=0, vertex.label.dist=2,
       vertex.label=NA, vertex.color=Ecolors,
```

```
       canvas.width = 1000, canvas.height = 1000)

Ecolors <- rep(c(col[1], col[5]), c(79, 19))
G1<-graph_from_adjacency_matrix(as.matrix(Sep.Net[[2]][[1]]), mode="undirected")
tkplot(G1, vertex.size=4,vertex.label.cex=0, vertex.label.dist=2,
       vertex.color=Ecolors,
       canvas.width = 1000, canvas.height = 1000)

Ecolors <- rep(c(col[1], col[6]), c(152, 15))
G1<-graph_from_adjacency_matrix(as.matrix(Sep.Net[[3]][[1]]), mode="undirected")
tkplot(G1, vertex.size=4,vertex.label.cex=0, vertex.label.dist=2,
       vertex.label=NA, vertex.color=Ecolors,
       canvas.width = 1000, canvas.height = 1000)

Ecolors <- rep(c(col[5], col[6]), c(20, 24))
G1<-graph_from_adjacency_matrix(as.matrix(Sep.Net[[4]][[1]]), mode="undirected")
tkplot(G1, vertex.size=4,vertex.label.cex=0, vertex.label.dist=2,
       vertex.color=Ecolors,
       canvas.width = 1000, canvas.height = 1000)

Ecolors <- rep(c(col[2], col[6]), c(378, 38))
G1<-graph_from_adjacency_matrix(as.matrix(Sep.Net[[5]][[1]]), mode="undirected")
tkplot(G1, vertex.size=4,vertex.label.cex=0, vertex.label.dist=2,
       vertex.label=NA, vertex.color=Ecolors,
       canvas.width = 1000, canvas.height = 1000)

Ecolors <- rep(c(col[3], col[5]), c(2, 14))
G1<-graph_from_adjacency_matrix(as.matrix(Sep.Net[[6]][[1]]), mode="undirected")
tkplot(G1, vertex.size=4,vertex.label.cex=0, vertex.label.dist=2,
       vertex.color=Ecolors,
       canvas.width = 1000, canvas.height = 1000)

Ecolors <- rep(c(col[3], col[6]), c(2, 15))
G1<-graph_from_adjacency_matrix(as.matrix(Sep.Net[[7]][[1]]), mode="undirected")
tkplot(G1, vertex.size=4,vertex.label.cex=0, vertex.label.dist=2,
       vertex.color=Ecolors,
       canvas.width = 1000, canvas.height = 1000)

Ecolors <- rep(c(col[1], col[4], col[5]), c(2, 1, 17))
G1<-graph_from_adjacency_matrix(as.matrix(SubNetworkDat), mode="undirected")
tkplot(G1, vertex.size=4,vertex.label.cex=0, vertex.label.dist=2,
       vertex.color=Ecolors,
       canvas.width = 1000, canvas.height = 1000)

Ecolors <- rep(col, c(158, 378, 2, 69, 19, 40))
G1<-graph_from_adjacency_matrix(as.matrix(CityDat[[1]]), mode="undirected")
tkplot(G1, vertex.size=4,vertex.label.cex=0, vertex.label.dist=2,
       vertex.label=NA, vertex.color=Ecolors,
       canvas.width = 1000, canvas.height = 1000)
```
