# Supplementary figures and images for: Identification of potential vulnerable points and paths of contamination in the Dutch broiler meat trade network
Source: PLoS One. 2020 May 15;15(5):e0233376. doi: 10.1371/journal.pone.0233376 (PMC7228058; doi:10.1371/journal.pone.0233376)

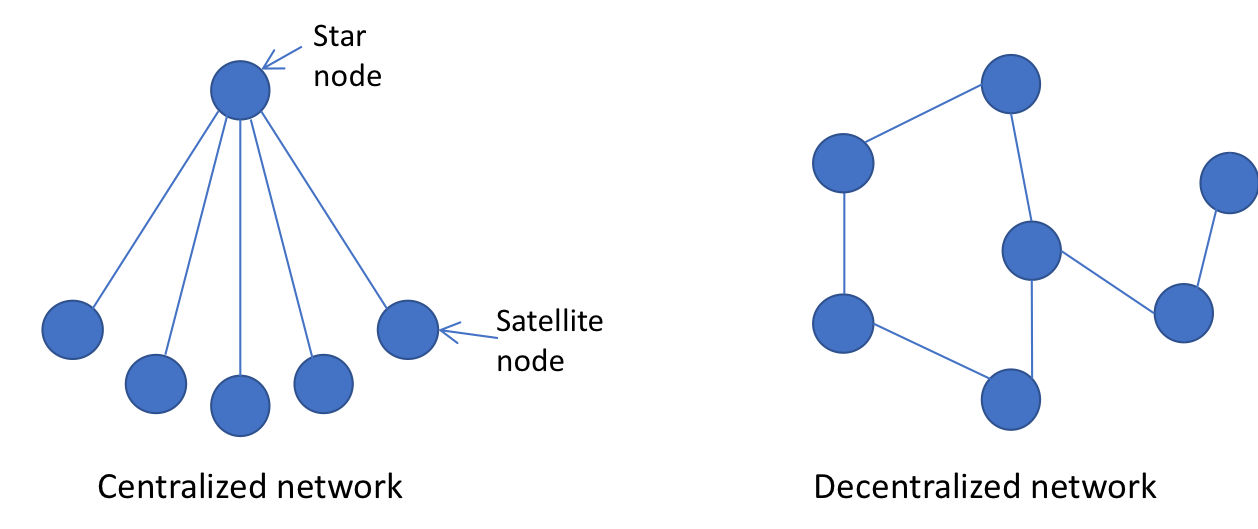

Supplement: S1 Fig — (TIF) [file pone.0233376.s003.tif]

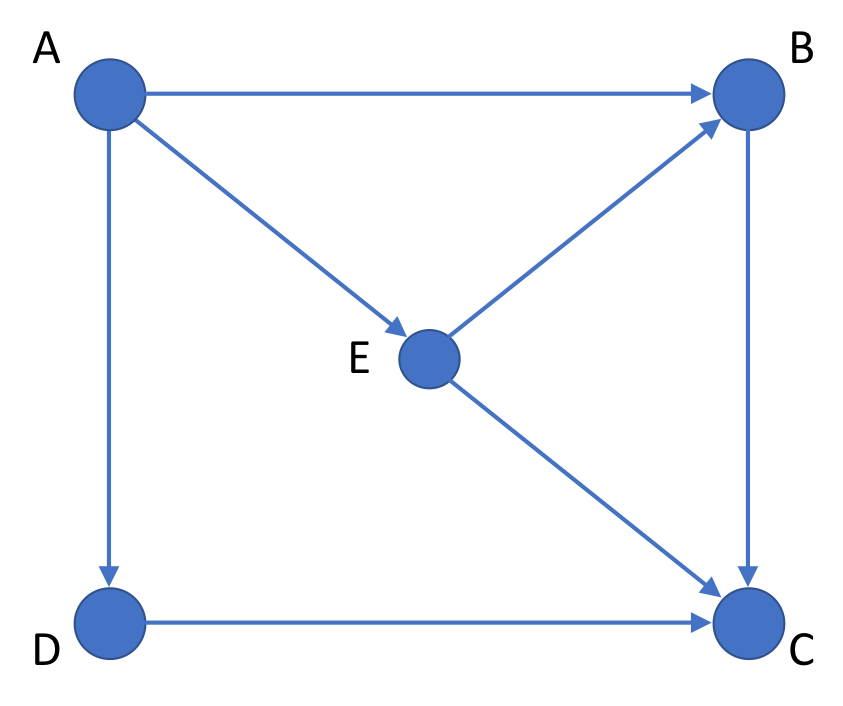

Supplement: S2 Fig — (TIF) [file pone.0233376.s004.tif]
