# Supplementary material for: Identification of potential vulnerable points and paths of contamination in the Dutch broiler meat trade network
Source: PLoS One. 2020 May 15;15(5):e0233376. doi: 10.1371/journal.pone.0233376 (PMC7228058; doi:10.1371/journal.pone.0233376)
Supplement: Attachment — Submitted filename: PONE-D-20-02018.doc [file pone.0233376.s005.doc]

The authors have built the track-and-trace system in Dutch broiler meat using graph theory to identify the potential contamination sources and critical paths. The manuscript is well structured and reports a very interesting study with valuable results, and some changes are suggested.

Minor comments:

Line 7: Number of affiliation is wrong

Line 20: modeled (UK past participle) should be changed to modeled (USA past participle)

Line 22: sub networks should be changed into sub-networks all through the manuscript.

Line 329: Pinior et al.: you miss the no. of reference

-The discussion needs to be streamlined and better ordered
